# An adaptive-neuro fuzzy inference system based-hybrid technique for performing load disaggregation for residential customers

**DOI:** 10.1038/s41598-022-06381-7

**Published:** 2022-02-11

**Authors:** Muhammad Zaigham Abbas, Intisar Ali Sajjad, Babar Hussain, Rehan Liaqat, Akhtar Rasool, Sanjeevikumar Padmanaban, Baseem Khan

**Affiliations:** 1 Department of Electrical Engineering, University of Engineering and Technology Taxila, Taxila, 47050 Pakistan; 2 Department of Electrical Engineering, Pakistan Institute of Engineering & Applied Sciences, Islamabad, 45651 Pakistan; 3 Department of Electrical Engineering, Sharif College of Engineering and Technology, Lahore, 55150 Pakistan; 4 Department of Electrical and Electronics Engineering, Center for Electric Vehicles and Power, Anna University, Chennai, India; 5 Department of Electrical and Computer Engineering, Hawassa University, Hawassa, Ethiopia

**Keywords:** Electrical and electronic engineering, Energy infrastructure

## Abstract

Effective and efficient use of energy is key to sustainable industrial and economic growth in modern times. Demand-side management (DSM) is a relatively new concept for ensuring efficient energy use at the consumer level. It involves the active participation of consumers in load management through different incentives. To enable the consumers for efficient energy management, it is important to provide them information about the energy consumption patterns of their appliances. Appliance load monitoring (ALM) is a feedback system used for providing feedback to customers about their power consumption of individual appliances. For accessing appliance power consumption, the determination of the operating status of various appliances through feedback systems is necessary. Two major approaches used for ALM are intrusive load monitoring (ILM) and non-intrusive load monitoring (NILM). In this paper, a hybrid adaptive-neuro fuzzy inference system (ANFIS) is used as an application for NILM. ANFIS model being sophisticated was difficult to work with, but ANFIS model helps to achieve better results than other competent approaches. An ANFIS system is developed for extracting appliance features and then a fine tree classifier is used for classifying appliances having more than 1 kW power rating based on the extracted feature. Several case studies have been performed using ANFIS on a publicly available United Kingdom Domestic Appliance Level Electricity (UK-Dale dataset). The simulation results obtained from the ANFIS for NILM are compared with relevant literature to show the performance of the proposed technique. The results prove that the novel application of ANFIS gives better performance for solving the NILM problem as compared to the other existing techniques.

## Introduction

Across the globe, energy is considered as one of the most important aspects for growth and development of the industrial, commercial sector and as well as a part of the residential area for energy conservation. Effective and efficient use of energy is a key point in energy sustainable development. With the rising cost and depleting sources of energy, the importance of energy consumption has become a hot topic of research in recent times. ALM techniques are important for determining the operational status of various appliances of a utility network. In general, the major goal of ALM is to identify and measure the power consumption of residential customers as well as the utility grid. Load measurements in the system help to determine the appliance operating status and energy consumption of each appliance. ALM involves the study of the appliance usage behavior for the conservation of energy by providing detailed feedback on appliance power consumption to consumers^1^. The recent development in the field of computer studies, measuring instruments, and communication technologies, has facilitated the application and working of ALM over a large number of residential, and industrial, and commercial consumers^1^.

Two major approaches used for ALM are ILM and NILM. ILM involves the installation of special measuring sensors with individual appliances. Generally, ILM is classified into two types: (i) direct method, (ii) indirect method. In the direct method, sensors installed with the appliance, directly measure the electrical characteristic of each appliance’s power demand whereas, the indirect method involves the installation of sensors with appliances that measure the non-electrical characteristics which are transformed into electrical characteristics later in Ref.^2^. The direct method for monitoring is further classified into three categories, i.e., sub-metering, smart appliances, and electric probing, whereas indirect monitoring includes appliance tagging, ambient sensors, and conditional demand analysis (CDA)^2^.

**Figure 1:**
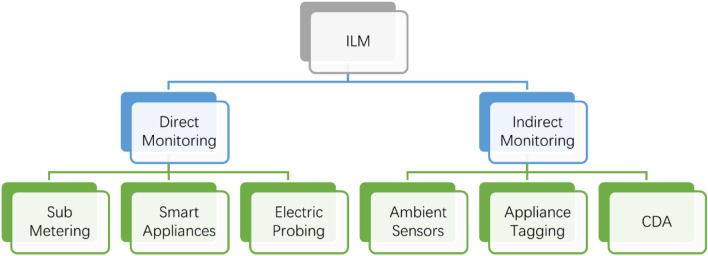
Intrusive load monitoring types.

Although being accurate, ILM is an expensive technique as it involves the installation of many sensors. Moreover, regular maintenance of these sensors is also required to ensure their accurate working. These considerations make ILM a secondary technique for load monitoring. Different types of ILM are shown in Fig. 1.

ILM framework can easily be understood by three stages^3^:

- *Appliance detection phase* This phase includes the use of one of the above-discussed ILM types for detecting the ON or OFF status of the appliance.
- *Interpretation phase* This phase includes software that interprets the appliance status received from the appliance detection phase.
- *Appliance status detection phase* The last phase of ILM determines the operating status for control and monitoring.

NILM does not require the installation of a smart appliance, nor any other intrusive technique for load monitoring. It can be described as a process for disaggregation of appliance power consumption gathered at the main measuring point making it a cost-effective and reliable technique in the field of ALM.

NILM was introduced in the late 1980s by Hart^4^. Hart in his algorithm studied the power signature of certain appliances within the smart meter. During his research, Hart alongside NILM has also discussed various types of appliances. The devices can be classified into the following categories:

i. ON/Off appliances, such as lamps and toasters having just two states, are categorized as Type 1 appliances.
ii. Multi-state devices, for example, washing machines also known as finite state machines are kept in Type 2 appliances.
iii. Devices, like drill machines and fan regulators that draw continuously varying power throughout their operation-known as continuously varying devices, are categorized as Type 3 appliances.

Later, a new category of appliances that are active throughout time, for example, telephone sets and internet routers are known as permanent consuming devices, is discovered and categorized as Type 4 appliances^5,6^.

NILM framework can easily be understood by the three-stage process:

- *Data acquisition* For NILM data acquisition is done by collecting the aggregate demand from the smart meter. The data from the smart meter either can be sampled at a high frequency or a low sampling rate. A low sampling rate can help to extract the profile of appliances with a higher power rating and a high sampling rate can help for extracting the profile of low power rating appliances^2^.
- *Feature extraction* Load features (more commonly termed as load signatures) are defined as the measurable parameters of aggregate load that provide information regarding the operating status and nature of working appliances^7^. Load features are further classified into three categories^8^:
  i. *Steady-state features* These features are extracted when the appliance is working in a steady-state. Steady-state features include steady-state power, power factor, RMS voltage, and currents.
  ii. *The transient state features* The features extracted in the period between ON time to steady-state are known as transient state features. These features include sudden circuit changes and are reliable when working with individual appliances as the transient features for each appliance are distinct.
  iii. *Non-traditional features* Parameters like temperature, start time, peak time, day, and light-sensing are non-traditional features.
- *Load identification* The last phase for NILM is load identification. Features extracted for appliances are used for algorithms that identify loads. Load identification can be further divided into two categories which include supervised and unsupervised learning.

Recently fuzzy logics and ANFIS have been used and tested in application for energy management and estimation. Linear quadratic rectangular based on fuzzy logics (LQRF) has been developed for variable speed variable pitch wind turbine. This model was used for evaluating optimal performance of controller on respective problem^9^. AN ANFIS model was developed recently for modelling climate change impact on wind power^10^. A hybrid Enhanced Elephant Herding Optimization Algorithm (EHOA) and ANFIS collectively known as (EHO-ANFIS) have been developed for modelling of microgrid and optimal allocation of low cost grid^11^. Looking at the potential of ANFIS, the following contribution is made in this paper:

1. Development and use of ANFIS novel application on NILM.

**Figure 2:**
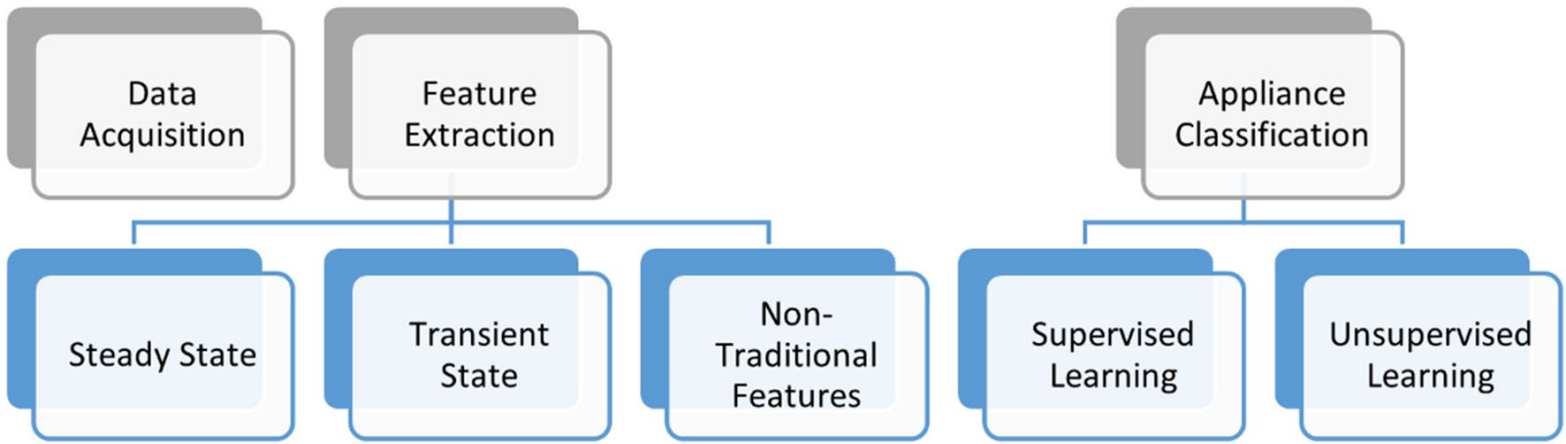
Framework for NILM.

The framework of the NILM is shown in Fig. 2.

The rest of the paper is arranged as follows. The literature review is included in “Literature review” section. The methodology is explained in “Methodology” section. The results and their analysis are described in “Results and discussion” section. “Conclusion” section includes the conclusion and recommendations for future work.

## Literature review

As discussed earlier, the appliance features through NILM can be extracted in three ways that include steady-state, transient state analysis, and non-traditional features. Steady state-based NILM uses active power (P) and reactive power (Q) features derived by identifying ON/OFF events of the appliance to extract appliance features^4^. The steady-state operation also depends upon the type of load/appliance. For the case of resistive load in which both current and voltage remain in phase, only the active power feature is considered. But in the case of inductive load, the current and voltage of load are out of phase. Therefore, both active and reactive power is used for extracting appliance features.

Disaggregation of load using only active power proved successful in the case of high-power-consuming appliances like electric heater and kettle, etc., as these appliances have a distinct operational state and have less complex power signatures^12–14^. However, appliances sharing common power signatures make appliance feature extraction difficult by using active power only. Similarly, simultaneous activation of the appliance also causes a problem for identifying appliances using active power.

Issues related to simultaneous appliance activation and common power signatures are analyzed and sorted by observing the step-change in active and reactive power of high-power rated Type-I and Type-II appliances. By observing the step-change in power, these appliances are distinguished easily. But, if some appliances at some instant, have overlapping P-Q characteristics, then it becomes hard to distinguish between the profile of appliances^12^. In Refs.^15–17^, the researchers have focused/switched towards the analysis of current and voltage profiles extracted from the appliance profile for avoiding the issues related to steady-state power change feature extraction. Each appliance profile possesses a unique root means square (RMS) and peak currents and voltages, a phase difference, and power factor. These parameters jointly build an appliance profile. When these V-I trajectory-based techniques are applied for real-time appliance recognition and profiling (RECAP), they showed promising performance, especially for type-I appliances. In Refs.^17,18^ the authors have successfully classified a group of appliances using a V-I trajectory-based method. During his research, he plotted the V-I trajectory using normalized current and the voltage value of a certain appliance. V-I trajectory enabled the authors to divide appliances into certain groups with high accuracy. However, V-I trajectory-based techniques lack the operation of multi-state appliance activation as in case multi-state devices, current, and voltage do not remain the same for each cycle operational state.

Discussion and experimental demonstration about the recognition of various loads through time-domain features analysis are done in Ref.^19^. At the end of this research, it is concluded that the RMS features are more accurate and reliable for feature extraction as compared to peak parameter features. However, in the said literature, the authors did not discuss the simultaneous activation of appliances and experimentation has been done only for type-III appliances.

In Refs.^20–22^, it has been revealed through experimentation that features of load having constant power and load with constant impedance can be extracted by observing the input harmonics current. Appliance features through Fourier transform are found out by analyzing the proportion of harmonic current between constant impedance and constant power load by decomposing the power consumption of the appliance. The current drawn by the non-linear load is non-sinusoidal, therefore, the non-linear load can easily be identified by using Fourier transform. However, a high sampling rate is required for extracting harmonic current waveforms using this technique. Estimation of appliance signal through current harmonics is most suitable for type-I and type-IV appliances.

NILM by transient analysis is more distinctive as compared with steady-state analysis because each appliance in the transient state has fewer overlapping characteristics. However, to perform a better NILM study by transient analysis, a high sampling rate is required^8^. Transient event shape can also be used for extracting the appliance features and classification^12^. The authors in Ref.^20^ have utilized overshoot power spikes of transient events of appliances as a component to distinguish appliance features. The downside of utilizing an overshoot power spike for feature extraction is that this strategy is appliance specific. This technique may not perform under simultaneous appliance operation and require a high sampling rate for proper performance. Table 1 shows different sampling rates used in literature for feature extraction of appliances along with the appliances identified at a specific sampling rate.

**Table 1:** Data sampling rate and appliance identification^31,32^.

Data Sampling frequency	Features	Appliance identification
1 s–1 min	Steady-state transition	Major appliances like a kettle, shower, etc
15 min–1 h	Average consumption time	None
kHz	Low or medium order harmonics	Minor appliances (TV and computers)
MHz	Higher-order harmonics	More than 20 minor appliances including lightening load

Optimization-based techniques for NILM are discussed in Refs.^23,24^. In Ref.^25^, the authors have solved the NILM problem using segmented quadratic integer constraint programming. Supervised learning like neural networks, support vector machines, and deep learning has been vastly used for solving the NILM problem^26–30^. Supervised learning requires a labeled data set of appliances for training. This trained dataset is then used to identify and extract the features of the appliances. In Ref.^26^ the authors have used a deep long short-term memory (LSTM) for extracting the features of appliances and classifying appliances in sets.

A deep dictionary and deep transform-based deep learning technique are proposed in Ref.^27^. A deep convolution neural network has been proposed in Ref.^28^ for data reinforcement with the requirement of sub-metering for unseen household datasets. It is a post-processing technique.

In unsupervised learning algorithms, no labeled data is required. Unsupervised learning algorithms are responsible for the collection of features of the appliances through the power consumption dataset^33,34^.

A thorough discussion about some recent trends mentioned above makes it clear that numerous techniques have been applied for improving the results of NILM but there is still improvement required for getting NILM results closer to ILM for the future of sustainable energy.

In this paper, a novel hybrid technique is proposed for improving the accuracy of NILM. The high-power rating appliances are disaggregated through an adaptive neuro-fuzzy inference system. The proposed method uses a neuro-fuzzy inference system for training labeled data for extracting appliance features. The proposed method is applied to low-frequency data from the UK-Dale dataset^35^. Some other potential alternative other than ANFIS for NILM on low or high frequency data maybe are Neural Networks (NN), Graph Signal Processing (GSP), Dynamic Programming (DP) and Linear Programming (LP).

## Methodology

**Figure 3:**
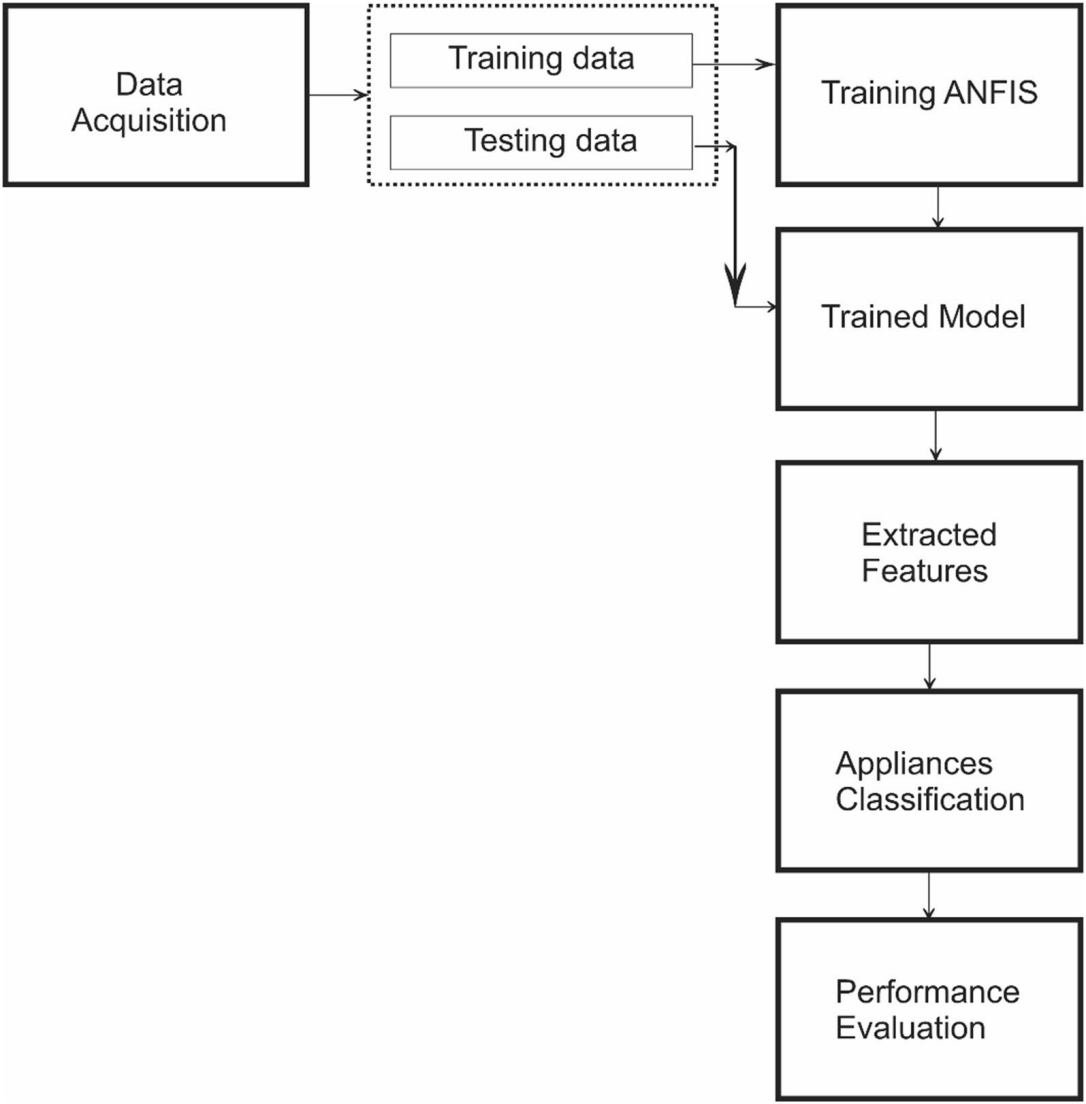
Proposed methodology flowchart.

The proposed methodology consists of a series of steps which include a selection of dataset followed by database creation, development of an adaptive neuro-fuzzy inference system, feature extraction, and appliance classification. The flowchart for the proposed algorithm is shown in Fig. 3 and each block of this flowchart is explained in the following subsections.

### Dataset

Data acquisition is generally defined as the process of measuring any physical or electrical quantity that can be voltage, current, frequency, power factor, and active and reactive power. The data acquisition system consists of a sensor for measuring the electrical quantity and a smart meter to process the sensor data to the user.

A load of any residential, commercial, or industrial sector at any time is given by:

1 $$P\left(t\right)=\sum_{i=1}^{n}{P}_{i}\left(t\right),$$

where ${P}_{i}\left(t\right)$ is the consumption of ith appliance at the time ‘$t$’ and ‘$n$’ is the total number of appliances.

NILM requires data from individual appliance sensors as well as aggregate demand. Several datasets are available publicly for carrying out research and validation of proposed methodologies as given in Table 2. In this paper, we have used the UK-Dale dataset available publicly^35^.

**Table 2:** Publicly available dataset for NILM.

Dataset	Institute	Location	Number of houses	Duration per house	Aggregate sampling time	Appliance sampling time
BLUED^37^	CMU	PA, USA	1	8 days	8 ms	N/A
REDD^38^	MIT	MA, USA	6	3–19 days	1 s	3 s
IHEPCD^8^	EDF	France	1	4 years	1 min	N/A
Sample dataset^39^	Pecan Street	TX, USA	10	7 days	1 min	1 min
AMPDs^40^	Simon Fraser	Vancouver, Canada	1	1 year	1 min	1 min
UK-Dale^40^	Imperial College	London, UK	5	3–17 months	6 s	1–6 s

UK-Dale dataset of NILM is an open-access dataset that is sampled at 16 kHz for measuring aggregate demand. For individual appliances, the sampling rate is 1/6 Hz. The dataset consists of aggregate and appliance level data of 6 residential houses in the UK.

Choosing UK-Dale dataset over other dataset have several advantage which first of all is variety of data available over a large time period for several user, the other main advantage of selecting this dataset over other is the public access and sampling rate.

### Database creation

In deep learning projects, the data is divided into training, testing, and validation data. Training datasets are used to train models that perform various actions on developed deep learning models.

The training dataset, which is used to train the algorithm, includes both inputs and the expected output. While test data is used to evaluate how well your data is trained during the training phase. In our scenario, we have used 70% of our data as training while 30% of data is kept as testing data as discussed earlier. Moreover, ANFIS has been used for evaluating the performance of the defined NILM problem.

### Adaptive neuro-fuzzy inference system (ANFIS)

Neural networks and fuzzy interface systems may be combined to make an ANFIS to compensate for the disadvantages of each other^41^. ANFIS is a learning technique that transforms inputs to output through fuzzy logic and highly interconnected neural networks. ANFIS uses the neural network training parameters to tune the parameters of the fuzzy inference system. The features that make ANFIS a commendable technique for achieving goals is:

i. It defines the behaviour of a complex problem/system by refining the IF-ELSE rule.
ii. It is easy to develop with no prior expertise required.
iii. It has the capability of supporting both numeric and linguistic knowledge.

**Figure 4:**
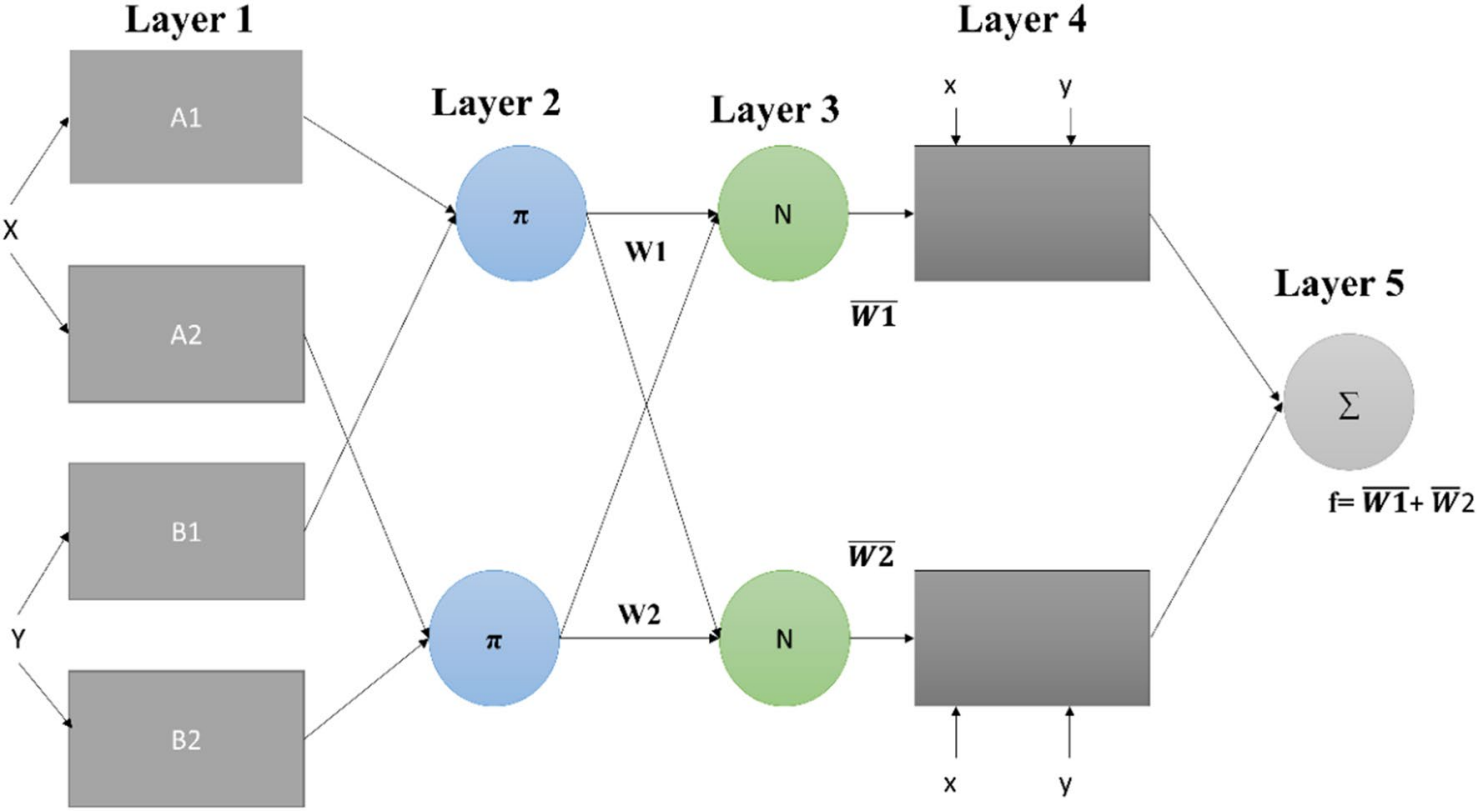
ANFIS model.

In ANFIS, every output should have its membership function. No rule is shared by more than one output. So in ANFIS, the number of rules must be equal to the number of membership functions^42^. The basic ANFIS model developed is shown in Fig. 4. In this Figure, fixed nodes and adaptive nodes are represented by the circles and the squares, respectively. The structure contains five layers named:

- Fuzzification layer.
- Rule layer.
- Normalization layer.
- Defuzzification layer
- Output layer.

The task for the fuzzification layer is to get the input values and determine the membership functions that belong to respective inputs. Standard inputs are transformed into fuzzy input in this layer. Layer 2 is responsible for generating the firing strength for the rule. The third layer is responsible for normalizing the computed firing strength by dividing each value by the total firing strength. Layer 4 takes the normalized input and in this inference of the system output for each rule is a linear combination of the input variable added up with a constant term. Values returned from layer 4 are defuzzified ones.

The values from layer 4 are processed to layer 5 to return the final output value. The final output value is the weighted average of each rule output. Output for each layer is given from Eqs. (2) to (8). Gaussian membership function for layer 1 is given as:

2 $${out}_{1,i}={\mu }_{Ai}\left(x\right) i=\mathrm{1,2},$$

3 $${out}_{1,i}={\mu }_{Bi-2}\left(y\right) i=\mathrm{3,4}.$$

As shown in Fig. 4, $A$ and $B$ are representing linguistic labels and $x$ and y are inputs with the node being represented as *i*. Node function ${\mu }_{Ai}$ and ${\mu }_{Bi-2}$ can be adopted by any membership function such as gaussian function, such as given in (4)^43^;

4 $${\mu }_{Ai}(x)={e}^{{-\left(\frac{x-{c}_{i}}{ai}\right)}^{2},}$$

Here, $a$ *and* $c$ represent membership function parameters.

5 $${Out}_{2,i=}{\mu }_{Ai}\left(x\right)\times {\mu }_{Bi}\left(y\right),$$

6 $${Out}_{3,i=}\frac{{w}_{i}}{{w}_{1}+{w}_{2}},$$

7 $${Out}_{4,i=Wi} ({p}_{i}x+{q}_{i}y+{r}_{i)}),$$

8 $${Out}_{5,i=}\frac{\sum i {w}_{i}{f}_{i}}{\sum i {w}_{i}}.$$

Parameters used for ANFIS are given in Table 3.

**Table 3:** ANFIS parameters used in the proposed research.

Parameters	Values/type
Nodes	100, 200, 300
Layers	5
Epoch	20
Optimization method	Hybrid
Input membership function type	Gaussian membership function
Output membership function	Constant
FIS Structure	Sugeno
Number of membership function for each input	200

Parameters used for ANFIS were selected on the best results, increasing number of epoch, nodes and membership function types has high impact on the processing time. Increasing the nodes, epoch will certainly increase the execution time. Error used ANFIS prediction model were Root Mean Square Error (RMSE) and Mean Absolute Error (MAE).

**Figure 5:**
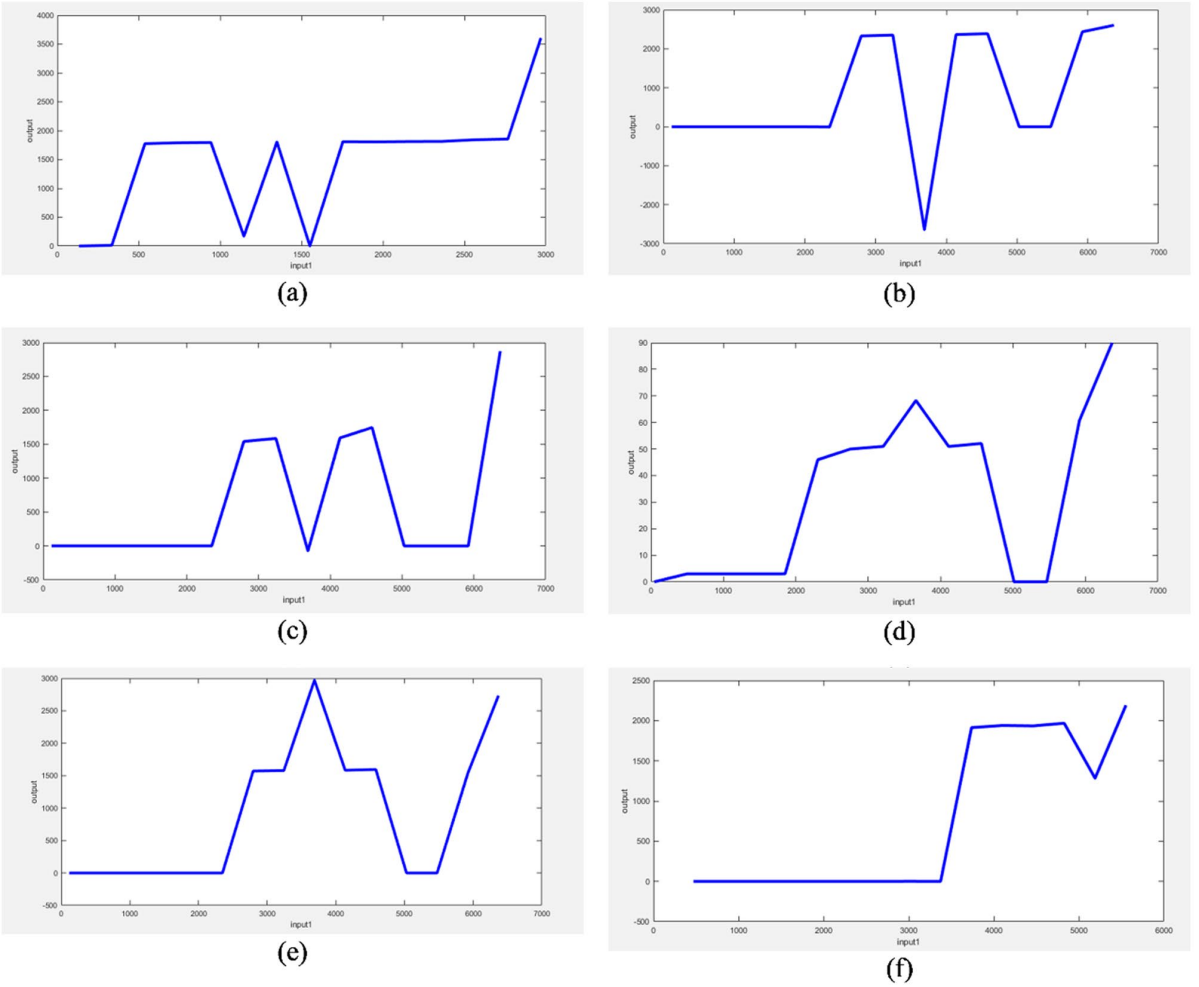
Post processing results of ANFIS (**a**) Iron, (**b**) Kettle, (**c**) Microwave, (**d**) Oven, (**e**) Toaster, (**f**) Vacuum Cleaner.

Post Processing results of ANIF are given in Fig. 5.

The post processing results of ANFIS shows corresponding in between input and output depending upon the membership function and fuzzy rules.

### Feature extraction

Every device present in the dataset contains a unique power signature and operational profile that is used to distinguish it from other active appliances. As mentioned earlier for database creation, we have used 30% of testing data and 70% of training data. Selection of above-mentioned data was selected because the mentioned proportion are practiced generally^44^. Once the data is trained and tested, the ANFIS is ready to extract the features of appliances.

**Figure 6:**
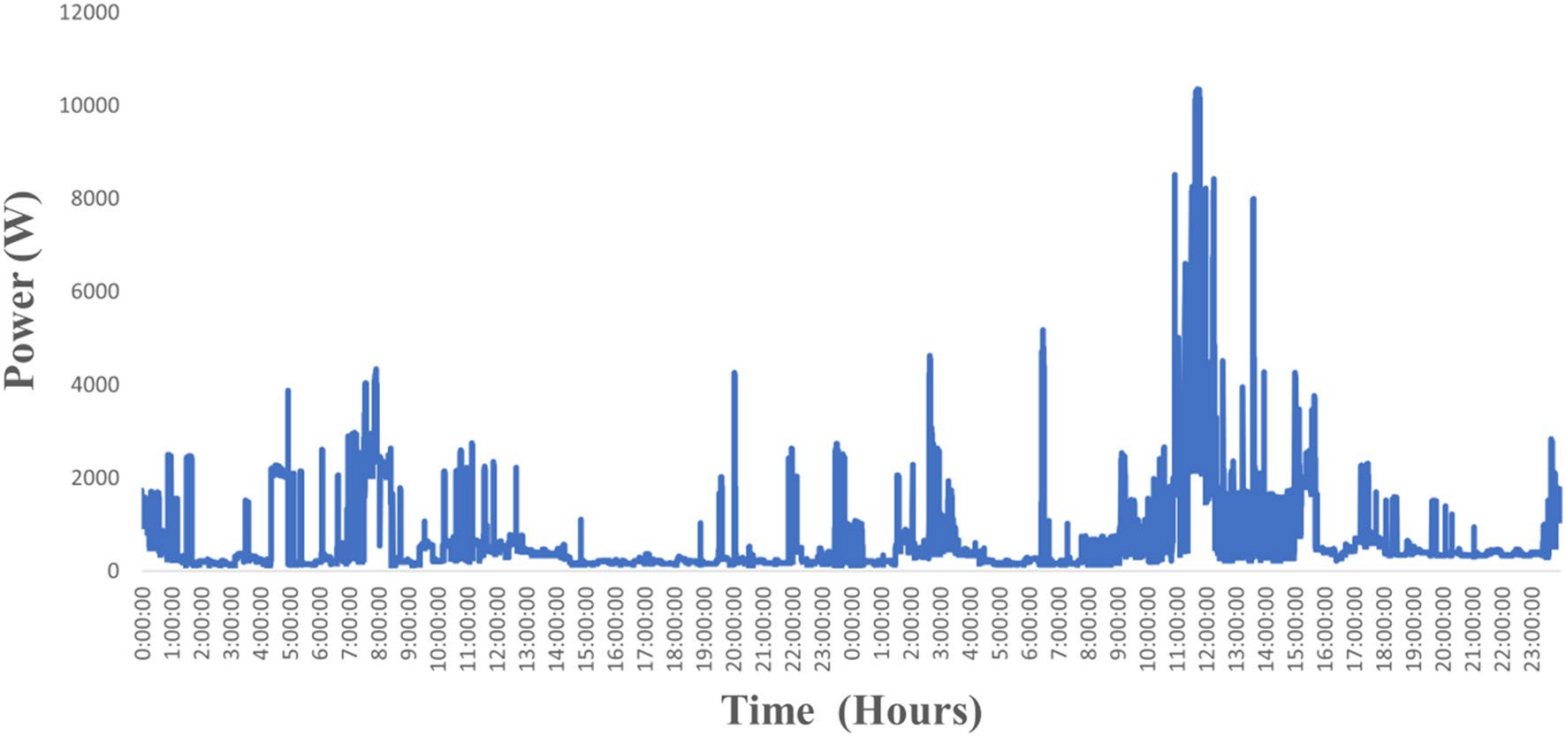
Aggregate Power consumption of a customer.

**Figure 7:**
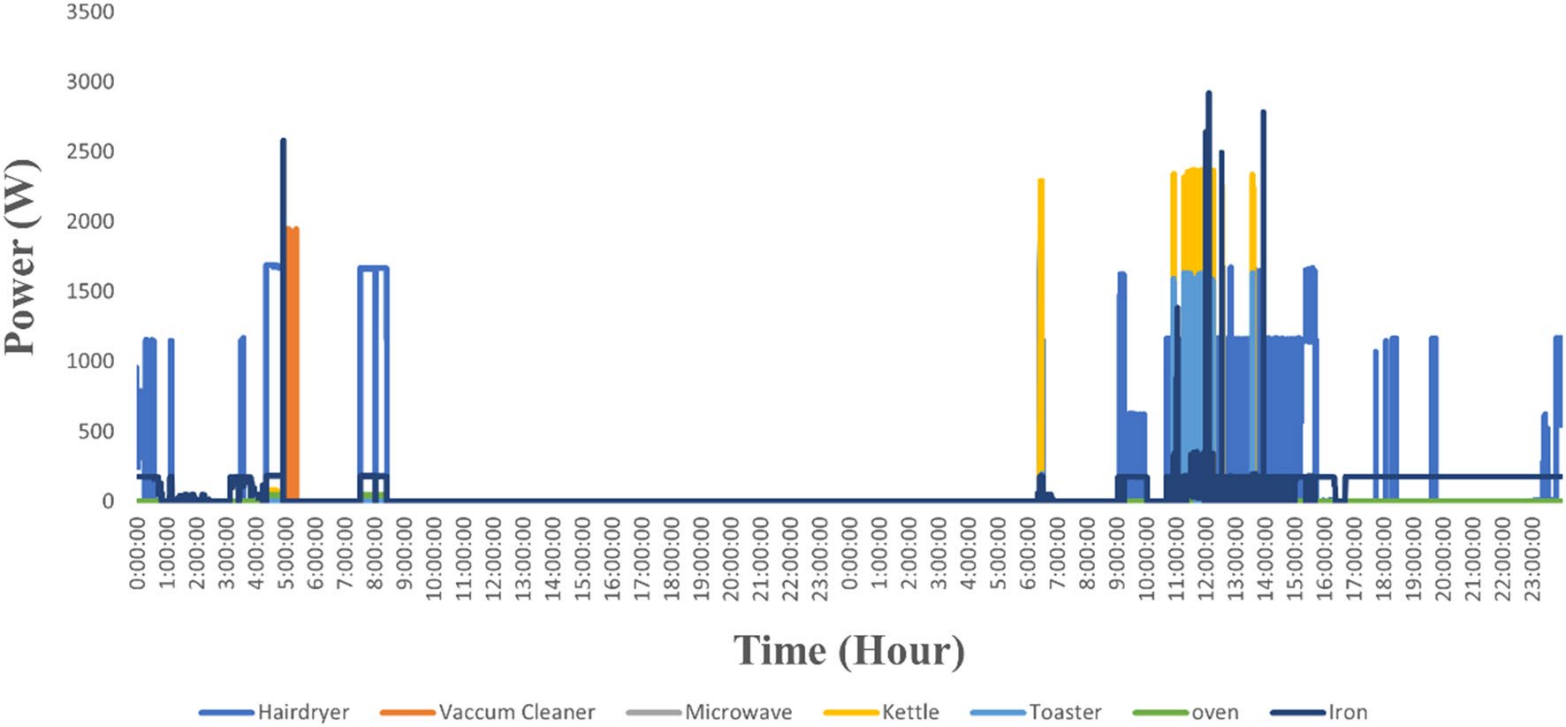
Extracted profile of individual appliance.

The appliance features are extracted through feeding aggregate demand as an input to ANFIS which is already trained and tested with appliance level data^45^. Figure 6 shows the aggregate demand for each ANFIS and the input parameter for every ANFIS. Figure 7 shows the appliance active power features that are extracted using the ANFIS model. The extracted feature of the appliance contains the active power consumption of the appliance at the time ‘t’. Once the appliance features are extracted the appliances can easily be classified using classifier learner (supervised learning).

### Appliance classification

The extracted feature of appliances, i.e., active power consumption is used for appliance classification. For the classification of appliances, a fine tree classifier leaner is applied. The fine tree classifier shows more effective results and maintains accuracy when compared to other machine learning techniques^46^. The performance of the proposed algorithm is measured based on parameters including precision (p), recall (r), and f1 score.

Precision defines whether the actual appliance activation is correctly classified by the algorithm.

Recall gives the probability that any appliance is detected by the algorithm whereas, harmonic mean between precision and recall shows the f1 score^36^.

The formula for calculating precision, recall, and f1 score is given in (9) to (11).

9 $$P=\frac{Tp}{Tp+Fp},$$

10 $$r=\frac{Tp}{Tp+Fn},$$

11 $$f1 score=2\times \frac{P*recall}{P+recall},$$

where *Tp* = No. of true positives, *Tn* = No. of true negative, *Fp* = No. of false positive, *Fn* = No. of false-negative.

The results for the proposed algorithm have been obtained using K-fold validations of threefold cross-validation, fivefold cross-validation, and sevenfold cross-validation. The several case studies discussed in the research are given in Table 4. K-fold cross-validation is used for the generalization of the model. In model training, sometimes the data get overfit, and to avoid this we use K-fold cross-validation to check how it is performing on test data. Moreover, K-fold cross-validation is also used to assess the predictive performance of the models and to check how they perform outside the sample to a new data set, also known as test data. K-fold validation work on specific setups which include:

- Shuffling of dataset randomly.
- Split dataset into K-folds/groups.
  I. Take the group as a test dataset.
  II. Take the rest of the group as the training set.
  III. Fit model on the training set and evaluate testing.

**Table 4:** Appliance classification case studies.

Dataset	House #	Sampling rate	Major appliances	Appliance type	ANFIS structure
UK-Dale	1	6 s	Kettle, microwave, oven, Hairdryer, iron, vacuum cleaner, and toaster	Type-I	5-layer 100 nodes
UK-Dale	1	6 s	Kettle, microwave, oven, Hairdryer, iron, vacuum cleaner, and toaster	Type-I	5-layer 200 nodes
UK-Dale	1	6 s	Kettle, microwave, oven, Hairdryer, iron, vacuum cleaner, and toaster	Type-I	5-layer 300 nodes

Evaluate the results using evaluation parameters.

The advantages of using above three, five, sevenfold cross validation was better results for precision, recall and f1 score as well as execution time. Other feasible solution may be a one, two, four, six, eight, ninefold cross validation which surely effect the execution time and might lower the precision, recall and f1 score which will be worst for real time application.

Focus of this research was on type-I appliances, therefore each case study included type-I appliances.

## Results and discussion

### Performance evaluation for different cases with different k-fold cross-validation

#### Performance evaluation for case 1 with different k-fold cross-validation

The performance evaluation results for case 1 are shown in Table 5. For the threefold cross-validation in case 1, the best performance is evaluated as 0.90, 0.87, and 0.88 for a toaster based on precision, recall, and f1 score, respectively while the model performed worst on performance for identifying kettle with having the value of 0.66 for precision, 0.66 and 0.68 for recall and f1 score respectively in case of the oven. For the fivefold cross-validation in case 1, the best performance is evaluated as 0.86, 0.86, and 0.86 for hairdryer based on precision, recall, and f1 score, respectively while the model performed worst on performance for identifying kettle with having the value of 0.61, 0.69 and 0.65 for precision, recall and f1 score, respectively. For the sevenfold cross-validation in case 1, the best performance is evaluated as 0.89, 0.86, and 0.86 for hairdryer, toaster respectively based on precision, recall, and f1 score, respectively while the model performed worst on performance for identifying kettle with having the value of 0.65 for precision, and 0.63 and 0.66 for recall and f1 score respectively in case of the oven. The precision results for case 1 show about the actual activation are also predicted as activation for respected appliances by the developed ANFIS model whereas the recall shows the results of any appliance activation detection by developed ANFIS model. 0.90 precision rate for toaster shows that from every 100 actual appliance activation 90 (90%) are detected correctly with the developed model. Further cases are also discussed in the coming section.

**Table 5:** Performance evaluation for case 1 with three, five, and sevenfold cross-validation.

Appliances	Threefold cross validation			Fivefold cross validation			Sevenfold cross validation		
	Precision	Recall	f1 score	Precision	Recall	f1 score	Precision	Recall	f1 score
Hair dryer	0.88	0.85	0.86	0.86	0.86	0.86	0.86	0.86	0.86
Sleeping	0.87	0.92	0.89	0.88	0.92	0.90	0.88	0.92	0.90
Unoccupied	0.86	0.75	0.80	0.86	0.76	0.81	0.86	0.76	0.81
Vacuum cleaner	0.74	0.76	0.75	0.79	0.67	0.73	0.78	0.73	0.75
Iron	0.82	0.87	0.84	0.84	0.85	0.84	0.85	0.85	0.85
Kettle	0.66	0.73	0.69	0.61	0.69	0.65	0.67	0.72	0.69
Microwave	0.76	0.68	0.72	0.76	0.7	0.73	0.75	0.71	0.73
Oven	0.71	0.66	0.68	0.79	0.69	0.74	0.69	0.63	0.66
Toaster	0.90	0.87	0.88	0.85	0.82	0.83	0.89	0.84	0.86
Average	0.8	0.79	0.79	0.80	0.77	0.79	0.80	0.78	0.79

#### Performance evaluation for case 2 with different k-fold cross-validation

The performance evaluation results for case 2 are shown in Table 6. For the threefold cross-validation in case 2, the best precision is found for the toaster as 0.87, the maximum recall is found for iron as 0.86 and the best f1 score is found for a hairdryer as 0.84 whereas, the worst performance of classification is for the oven in the case of precision, f1 score, and recall respectively having the value of 0.62, 0.53, and 0.57. For the fivefold cross-validation in case 2, the best precision was found for the toaster as 0.89 and the best recall was found for a hairdryer as 0.84, and f1 score was found best for the hairdryer as 0.85 while the model performed worst in case of precision and recall for a kettle with a value of 0.64 and 0.63 respectively and for recall the score was worst for oven with a value of 0.60. For the sevenfold cross-validation, the best precision was found for a toaster having a value as 0.90 and best recall for hairdryer and toaster as 0.84 while best f1 score was found for hairdryer and toaster having value 0.87 while kettle has the worst precision of 0.66 and worst recall and f1 score was found for the oven.

**Table 6:** Performance evaluation for case 2 with three, five, and sevenfold cross-validation.

Appliances	Threefold cross validation			Fivefold cross validation			Sevenfold cross validation		
	Precision	Recall	f1 score	Precision	Recall	f1 score	Precision	Recall	f1 score
Hair dryer	0.86	0.83	0.84	0.87	0.84	0.85	0.86	0.84	0.85
Sleeping	0.88	0.90	0.89	0.87	0.91	0.89	0.87	0.91	0.89
Unoccupied	0.81	0.76	0.78	0.82	0.75	0.78	0.83	0.74	0.78
Vacuum cleaner	0.74	0.78	0.76	0.71	0.71	0.71	0.75	0.80	0.77
Iron	0.79	0.86	0.82	0.80	0.85	0.82	0.80	0.85	0.82
Kettle	0.64	0.63	0.63	0.64	0.63	0.63	0.66	0.71	0.68
Microwave	0.63	0.71	0.67	0.67	0.70	0.68	0.69	0.66	0.67
Oven	0.62	0.53	0.57	0.74	0.60	0.66	0.73	0.59	0.65
Toaster	0.87	0.79	0.83	0.89	0.80	0.84	0.90	0.84	0.87
Average	0.76	0.75	0.76	0.78	0.754	0.77	0.79	0.77	0.78

#### Performance evaluation for case 3 with different k-fold cross-validation

The performance evaluation results for case 2 are shown in Table 7. For the threefold cross-validation in case 3, the best precision for toaster was found to be 0.90, the maximum recall was found for iron having a value of 0.86, and the best f1 score was found for toaster and hairdryer as 0.86 for each appliance whereas the worst performance of classification was for the kettle, vacuum cleaner in case of precision, f1 score, and recall, respectively. For the fivefold cross-validation in case 3, the best precision, recall, and f1 score was found for the hairdryer having a value of 0.87 whereas the classifier performed worst on microwave and kettle. The maximum recall was found for iron having a value of 0.86 and the best f1 score was found for the toaster and hairdryer as 0.86 for each appliance. For the sevenfold cross-validation, the best performing appliance was the toaster and hairdryer, and the worst performance of the classifier was on vacuum cleaner and oven.

**Table 7:** Performance evaluation for case 3 with three, five, and sevenfold cross-validation.

Appliances	Threefold cross validation			Fivefold cross validation			Sevenfold cross validation		
	Precision	Recall	f1 score	Precision	Recall	f1 score	Precision	Recall	f1 score
Hair dryer	0.86	0.86	0.86	0.87	0.87	0.87	0.87	0.87	0.87
Sleeping	0.90	0.91	0.90	0.90	0.91	0.90	0.90	0.91	0.90
Unoccupied	0.84	0.80	0.83	0.84	0.81	0.82	0.84	0.81	0.82
Vacuum cleaner	0.78	0.64	0.70	0.74	0.73	0.73	0.73	0.67	0.70
Iron	0.82	0.86	0.84	0.84	0.86	0.85	0.84	0.87	0.85
Kettle	0.65	0.71	0.68	0.63	0.71	0.67	0.68	0.73	0.70
Microwave	0.71	0.66	0.68	0.79	0.67	0.72	0.75	0.73	0.74
Oven	0.73	0.65	0.69	0.71	0.60	0.65	0.75	0.66	0.70
Toaster	0.90	0.83	0.86	0.81	0.83	0.82	0.89	0.80	0.84
Average	0.80	0.77	0.78	0.792	0.78	0.78	0.805	0.783	0.79

### Comparison of results

The proposed hybrid ANFIS technique of NILM was applied to the publicly available UK-Dale dataset. Different combination for ANFIS has been evaluated by changing the number of nodes and keeping layers constant. The model was evaluated for 5-layer-100 nodes, 5-layer-200 nodes, and 5-layer-300 nodes. Looking at the comparison of results mentioned in Table 8 we can see that the average value for each of the proposed research combinations has a better value for precision, and f1 score, whereas the value for recall proved to be better in the case of literature. The value of precision in the case of proposed case 1 (ANFIS with 100 nodes) lies between 0.61 and 0.90, the value of recall exists between 0.63 and 0.87, and the value of f1score lies in between 0.64 and 0.88. In case 2 (ANFIS with 200 nodes), the precision value lies between 0.62 and 0.90, the value of recall exists between 0.53 and 0.86, and the value of the f1score lies in between 0.57 and 0.86. In case 3 (ANFIS with 300 nodes), the value of precision lies between 0.63 and 0.90, the value of recall exists between 0.60 and 0.86, and the value of the f1score lies in between 0.65 and 0.86.

**Table 8:** Comparison of proposed cases with literature.

Cases	Precision (average)	Recall (average)	f1 score (average)
Proposed case 1 with threefold cross validation	0.8	0.78	0.79
Proposed case 1 with fivefold cross validation	0.8	0.77	0.78
Proposed case 1 with sevenfold cross validation	0.8	0.78	0.79
Proposed case 2 with threefold cross validation	0.76	0.75	0.76
Proposed case 2 with fivefold cross validation	0.77	0.75	0.76
Proposed case 2 with sevenfold cross validation	0.78	0.77	0.77
Proposed case 3 with threefold cross validation	0.79	0.76	0.78
Proposed case 3 with fivefold cross validation	0.79	0.78	0.78
Proposed case 3 with sevenfold cross validation	0.81	0.78	0.79
NN with edge detection^36^	0.559	0.89	0.65
NN^36^	0.763	0.857	0.776
LSTM-RNN^47^	0.778	0.752	0.764
LSTM^30^	0.36	0.85	0.38
Rectangles^30^	0.55	0.57	0.56
Autoencoder^30^	0.47	0.94	0.53
Factorial HMM^30^	0.12	0.53	0.18
Combinational optimization^30^	0.13	0.47	0.58

Whereas in the case of literature, the value of precision lies between 0.60–0.87, 0.64–0.99 for recall, and 0.66 to 0.89 for f1 score^36^. And for case 2, values for precision, recall, and f1score exist between 0.31–0.83, 0.73–0.99, and 0.36 to 0.90, respectively. The average value for literature is found to be 0.76, 0.85, and 0.776 for precision, recall and f1score respectively for case 1, and 0.551, 0.89, and 0.65 for precision, recall, and f1 score respectively for case 2. The average value for our case 1 was 0.80, 0.78, and 0.79 for precision, recall, and f1 score. The average value for our case 2 was 0.78, 0.77, and 0.78 for precision, recall, and f1 score. The average value for our case 3 was 0.80, 0.78, and 0.79 for precision, recall, and f1 score.

Looking at the percentage improvement taken from Tables 9, 10, 11, it can be seen that values of precision and f1 score are increased significantly in all proposed cases when compared with literature and outperforming the results given in paper^36^.

**Table 9:** Percentage improvement of proposed case 1 w.r.t base cases.

Average (percentage)	NN^36^	NN with edge detection^36^	The proposed technique for case 1	Percentage improvement w.r.t NN (%)	Percentage improvement w.r.t NN with edge detection (%)
Precision	76.3	55.9	80	4.625	26.7
Recall	85.7	89	78	–	–
f1 score	77.6	65	79	1.77	16.3

**Table 10:** Percentage improvement of proposed case 2 w.r.t base cases.

Average (percentage)	NN^36^	NN with edge detection^36^	The proposed technique for case 2	Percentage improvement w.r.t NN (%)	Percentage improvement w.r.t NN with edge detection (%)
Precision	76.3	55.9	78	2.18	28.3
Recall	85.7	89	78	–	–
f1 score	77.6	65	77	–	15.5

**Table 11:** Percentage improvement of proposed case 3 w.r.t base cases.

Average (percentage)	NN^36^	NN with edge detection^36^	The proposed technique for case 3	Percentage improvement w.r.t NN (%)	Percentage improvement w.r.t NN with edge detection (%)
Precision	76.3	55.9	81	5.80	31
Recall	85.7	89	78	–	–
f1 score	77.6	65	79	1.77	17.7

While in case of recall certain appliance showed improvement while some has not performed well as a result of which the average value for recall for proposed research has not improved significantly when compared to literature.

The best improvement we got is for 5-layers with 300 nodes, which means if nodes are increased the improvement can be further made in energy disaggregation. However, improving the nodes may take more time to execute the simulation.

Three parameters have been adopted as benchmark for evaluating performance and comparing proposed technique with literature. Other parameters that can also be used for evaluating performance are accuracy, positive predicted value (PPV) and negative predicted value (NPV). As the previous literature had majorly focused on precision, recall and f1 score therefor the parameters are used as benchmark for evaluating performance.

## Conclusion

In this paper, an adaptive technique for improved load disaggregation of a residential customer is developed. NILM has been applied for disaggregating the household data into appliance-level data. The proposed technique for NILM is a multilayer adaptive neuro-fuzzy inference system. ANFIS is used to extract the features of appliances and then, these features are used, for classifying the data into appliances using fine tree classifier learners. The threefold cross-validation, the fivefold cross-validation, and the sevenfold cross-validation are used for classifying the appliances. The proposed algorithm has been applied to major household appliances having a power rating greater than 1 kW.

The proposed technique is successful to detect simultaneous occurring events that have been rarely addressed in previous literature. Moreover, the proposed technique directly classifies the information during a time-series window, thus making it efficient and straightforward. The proposed ANFIS technique is a lot of time and memory consuming when compared with other approaches if the layers and epoch keeps on increasing.

In the future, this system may also be applied over type-II, III, and IV appliances. Alongside that, the accuracy of NILM may also be improved using reactive power with active power consumption.
